# Acantholytic pityriasis rubra pilaris associated with topical use of imiquimod 5%: case report and literature review^☆^^☆☆^

**DOI:** 10.1016/j.abd.2019.01.009

**Published:** 2019-11-23

**Authors:** Oriete Gerin Leite, Sandra Tagliolatto, Elemir Macedo de Souza, Maria Letícia Cintra

**Affiliations:** a Dermoclínica, Campinas, SP, Brazil; b Department of Dermatology, Universidade Federal de São Paulo, São Paulo, SP, Brazil; c Discipline of Dermatology, Department of Clinical Medicine, Universidade Estadual de Campinas, Campinas, SP, Brazil; d Department of Anatomical Pathology, Universidade Estadual de Campinas, Campinas, SP, Brazil

**Keywords:** Aminoquinolines, Drug eruptions, Keratosis, actinic, Methotrexate, Pityriasis rubra pilaris

## Abstract

Topical use of immune response modifiers, such as imiquimod, has increased in dermatology. Although its topical use is well tolerated, it may be associated with exacerbations of generalized cutaneous inflammatory diseases, possibly through the systemic circulation of pro-inflammatory cytokines. This report describes a case of development of pityriasis rubra pilaris, a rare erythematous-papulosquamous dermatosis, in a woman aged 60 years during treatment with imiquimod 5% cream for actinic keratosis. It evolved with erythrodermic conditions and palmoplantar keratoderma, presenting progressive clinical resolution after the introduction of methotrexate. The authors emphasize the importance of recognizing possible systemic reactions associated with the topical use of imiquimod.

## Introduction

Pityriasis rubra pilaris (PRP) is a rare erythematous papulosquamous dermatosis, clinically and pathologically similar to psoriasis. It has undetermined etiology, and although described as self-limiting, it can profoundly affect the patient's quality of life.

The association of PRP with the use of imiquimod in the treatment of actinic keratosis and superficial basal cell carcinoma has been reported in three articles. This report describes a case of PRP during the use of imiquimod for actinic keratosis, with a good response to treatment with methotrexate.

## Case report

**Figure 1:**
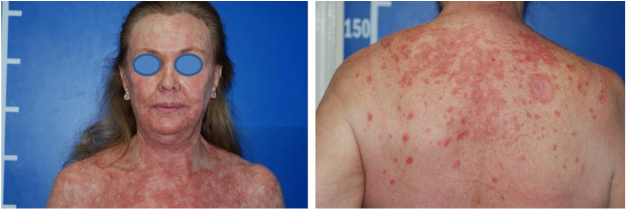
Eruptive erythematous papulosquamous lesions on the trunk and cephalic segment.

A 60-year-old Caucasian female patient had actinic keratosis for three months in the right chest, confirmed by biopsy. Imiquimod 5% cream was prescribed for topical treatment, five times per week. After two weeks, fever, diarrhea, myalgia, and fatigue associated with exulceration of the initial lesion and onset of erythematous papulosquamous lesions on the scalp, face, and trunk began (Fig. 1). Treatment was discontinued. Laboratory tests did not show any changes.

**Figure 2:**
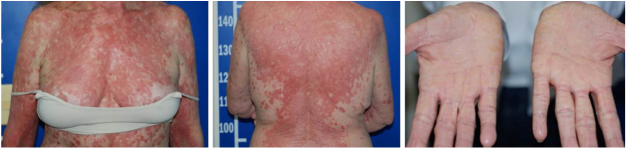
Confluence of the lesions and evolution to erythroderma, with areas of healthy skin. Note the palmar involvement with orange keratoderma.

**Figure 3:**
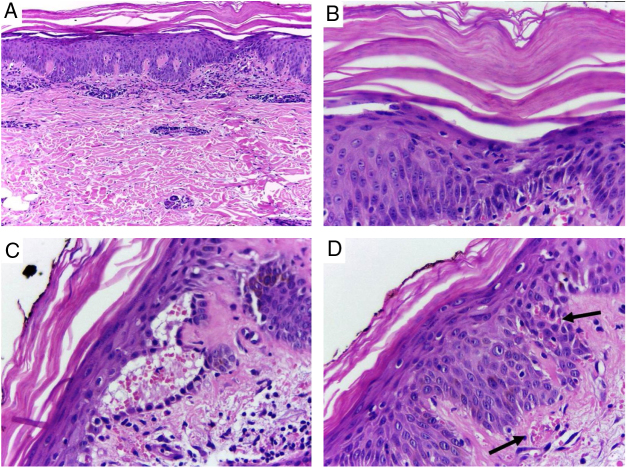
Skin, abdominal region, panoramic view: (A) hyperkeratosis, psoriasiform acanthosis, and moderate superficial perivascular lymphoid infiltrate; (B) detail of the previous image – there is altered hyperparakeratosis; (C) area of acantholytic dyskeratosis and supra-basal intraepidermal cleft; (D) marked congestion (arrow) with transepidermal elimination of red blood cells. Hematoxylin & eosin, original magnification of 100× (A) and 400× (B–D).

Despite the introduction of topical corticoids and oral antihistamines, there was worsening of the eruption with craniocaudal sense and confluence of the lesions (Fig. 2). Biopsy of the lesions was performed and prednisone was started, at 40 mg per day. In the histology, psoriasiform hyperplasia of the epidermis, hyperparakeratosis alternated with orthokeratosis, areas of hypogranulosis, moderate superficial perivascular lymphohistiocytic infiltrate, and foci of acantholytic dyskeratosis were observed (Fig. 3).

Four weeks after the onset of the eruption, the patient was erythrodermic, with areas of healthy skin associated with ectropion and orange palmoplantar keratoderma. The clinical–histopathological diagnosis of PRP was defined. Methotrexate was introduced orally at a dose of 15 mg per week and the oral corticosteroid was withdrawn gradually.

**Figure 4:**
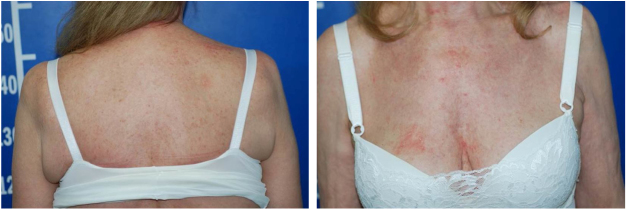
Disease resolution after nine months.

Serologic tests (HIV, hepatitis B and C) were negative and abdominal ultrasound and chest X-ray were normal. There was a progressive improvement in the erythrodermic condition after three months, when methotrexate reduction was started. The cutaneous surface was normalized nine months after the onset of the disease (Fig. 4).

## Discussion

PRP is a rare disorder of keratinization. The classic form (Type I) occurs in adults of both sexes, with eruption of follicular papules that converge in scaly erythematous-orange plaques, with craniocaudal spreading, usually evolving to erythroderma with healthy skin islets and palmoplantar keratoderma. Also described are atypical forms, the involvement of children, and a form associated with HIV infection.^1^

Histopathology shows psoriasiform hyperplasia, follicular hyperkeratosis, hyperparakeratosis alternated with orthokeratosis, and superficial perivascular lymphocytic infiltrate. The occurrence of acantholytic dyskeratosis, as in the present case, is common and may induce diagnostic errors due to histological similarity with Grover's, Darier's, or pemphigus.^2^ In 1997, Magro and Crowson suggested that the presence of acantholytic dyskeratosis is frequent and may serve as a predictive factor of PRP in the histopathological differentiation with psoriasis.^3^

Despite the unclear etiology, one of the main hypotheses for PRP is an exacerbated immune response to antigenic triggers. Viral, bacterial, neoplastic, and autoimmune diseases – as well as immune-modifying drugs – are believed to play a role in its pathogenesis.^1^, ^2^, ^3^ The T-helper 1 (Th1) immune response pathway is activated, with increased proinflammatory cytokines such as tumor necrosis factor-α, interferon-α, interleukin-1, interleukin-6, interleukin-17, and interleukin-23.^4^

Activation of the Th1 response would also be responsible for the poor vitamin A action found on the skin affected by PRP. Inflammatory cytokines alter the signaling of retinoid keratinocyte receptors, leading to abnormal keratinization and epidermal hyperplasia.^3^ Among the therapeutic options for PRP are oral retinoids, methotrexate, phototherapy, and biological agents with anti-tumor necrosis factor-α, anti-interleukin-17, and anti-interleukin-23 activity.^1^, ^2^

Imiquimod is an immune response stimulating agent, indicated for topical treatment of actinic keratosis, superficial basal cell carcinoma, and condyloma acuminatum. In recent years, its use has expanded to treat other off-label dermatological conditions.^5^

As a toll-like receptor-7 (TLR-7) agonist observed in the cells of the innate immune system (dendritic cells and macrophages) and keratinocytes, imiquimod performs antitumor and antiviral actions. Its binding to TLR-7 promotes increased production of Th1 proinflammatory cytokines such as tumor necrosis factor-α, IFN-α, interleukin-1, interleukin-6, and interleukin-8, among others, as well as inhibiting the anti-inflammatory pathway Th2.^5^

Its topical use is safe and well tolerated, with local and reversible transient reactions, but systemic adverse effects may occur. Flu-like symptoms, myalgia, fever, headache, and fatigue have been reported, reversible with medication suspension.^6^ More than the systemic absorption of imiquimod, the systemic circulation of inflammatory cytokines is credited with the etiology of these adverse effects.^6^

Imiquimod has been associated with the onset and exacerbation of inflammatory skin eruptions such as psoriasis, exfoliative dermatitis, erythema multiforme, pemphigus, and subacute lupus.^7^ Psoriasis has been induced by topical imiquimod in rats, with increased interferon-α and interleukin-1, interleukin-6, interleukin-17, and interleukin-23.^7^

In 2008, Yang et al. reported exacerbation of PRP in a 67-year-old patient under treatment with topical imiquimod 5% for actinic keratosis on the face and scalp. Two weeks after starting imiquimod, as in the present report, the patient developed an influenza-like illness associated with spreading of the follicular erythematous-desquamative lesions. There was improvement after 26 months of treatment with acitretin.^8^

In 2010, Gómez-Moyano et al. described erythrodermic PRP in a 56-year-old patient during treatment with imiquimod for superficial basal cell carcinoma on the back. As in the present case, the histopathology had foci of acantholytic dyskeratosis. There was improvement of PRP after two months with acitretin.^9^

Atanaskova Mesinkovska et al., in 2011, published a case of acantholytic PRP in a 65-year-old patient with topical use of imiquimod 3.75% for treatment of actinic keratosis. She presented improvement after narrowband ultraviolet B radiation phototherapy.^10^

The causal relationship between imiquimod and PRP remains unknown. Imiquimod may have been a trigger for PRP, as it promotes activation of the Th1 inflammatory pathway and the systemic cytokine circulation of tumor necrosis factor-α, interferon-α, corroborating the main theory for PRP pathogenesis.^1^, ^4^, ^5^ Flu-like systemic symptoms occurred after the use of imiquimod, without any evidence of an infectious process, which is an adverse effect that is already well documented.^5^, ^6^ In addition, the use of methotrexate, with antagonistic action upon tumor necrosis factor-α,^4^ resulted in regression of the lesions.

The use of immunologically active drugs in dermatology has been growing consistently. The dermatologist should be able to recognize possible systemic side effects and the exacerbation or outbreak of inflammatory skin diseases.

## Financial support

None declared.

## Authors’ contribution

Oriete Gerin Leite: Approval of the final version of the manuscript; conception and planning of the study; elaboration and writing of the manuscript; effective participation in research orientation; critical review of the literature.

Sandra Tagliolatto: Approval of the final version of the manuscript; conception and planning of the study; elaboration and writing of the manuscript; effective participation in research orientation; intellectual participation in propaedeutic and/or therapeutic conduct of the cases studied; critical review of the manuscript.

Elemir Macedo de Souza: Approval of the final version of the manuscript; intellectual participation in propaedeutic and/or therapeutic conduct of the cases studied; critical review of the literature; critical review of the manuscript.

Maria Letícia Cintra: Approval of the final version of the manuscript; elaboration and writing of the manuscript; intellectual participation in propaedeutic and/or therapeutic conduct of the cases studied; critical review of the literature; critical review of the manuscript.

## Conflicts of interest

None declared.
